# Case Report: Novel findings of larotrectinib in children with *NTRK*-rearranged spindle cell tumor

**DOI:** 10.3389/fonc.2025.1561051

**Published:** 2025-09-29

**Authors:** Linnan Wu, Weiji Xie, Juan Wang, Suying Lu, Yizhuo Zhang, Junting Huang

**Affiliations:** ^1^ Sun Yat-sen University Cancer Center; State Key Laboratory of Oncology in South China; Collaborative Innovation Center for Cancer Medicine, Guangzhou, China; ^2^ Department of Pediatric Oncology, Sun Yat-Sen University Cancer Center, Guangzhou, China

**Keywords:** larotrectinib, TRK inhibitor, *NTRK*-rearranged, children, spindle cell tumor

## Abstract

Neurotrophic tropomyosin receptor kinase (*NTRK*)-rearranged spindle cell tumors are often resistant to chemotherapy and radiotherapy. Fortunately, they are sensitive to targeted therapy of tropomyosin receptor kinase (TRK) inhibitors. However, the data on larotrectinib in Chinese children with *NTRK*-rearranged spindle cell tumor are still scarce. We reported 4 children with TRK fusion-positive solid tumors received larotrectinib in different clinical scenarios, including second-line treatment after progressive disease (patient #1), relapse after resection (patients #2 and #3), and metastatic disease (patient #4) and all of them benefited from the treatment. The patients harbored different TRK fusion genes (patient #1: TP53-*NTRK1*; #2: TPM3-*NTRK1*; #3: TPM3-*NTRK1*, DCST1-*NTRK1*, ZBTB7B-*NTRK1*, and *NTRK1*-DCST2; #4: LMNA-*NTRK1*). Our study provides new insights into the biology and management of *NTRK*-rearranged spindle cell tumors, contributing to the expanding evidence supporting the use of TRK inhibitors in these tumors. Further studies are needed to validate our findings and to explore the potential of second-generation TRK inhibitors in overcoming resistance.

## 1 Introduction

Neurotrophic tropomyosin receptor kinase (*NTRK*)-rearranged spindle cell tumors constitute a newly recognized group of soft tissue and bone tumors that are characterized by the fusion of *NTRK1, NTRK2 or NTRK3* genes with other genes, resulting in the overexpression of neurotrophic receptor tyrosine kinases proteins (1). *NTRK*-rearranged spindle cell tumors are often resistant to conventional chemotherapy and radiotherapy, with a high risk of local recurrence and distant metastasis (2). However, these tumors are sensitive to targeted therapy of tropomyosin receptor kinase (TRK) inhibitors, which have shown high response rates and durable effects in solid tumors with *NTRK* fusions.

TRK signaling plays a crucial role in the nervous system under normal physiological conditions (3). However, translocation of the NTRK gene with fusion partners (e.g., ETV6, LMNA, TPM3) generates a novel fusion oncoprotein that results in aberrant activation of TRK kinase, triggers downstream pro-oncogenic pathways (e.g., RAS/MAPKs, MAPK, PI3K/AKT/mTOR), and ultimately leads to unregulated cell proliferation (4–6). This translocation is common in rare pediatric tumors such as infantile fibrosarcoma (IFS) (7), congenital mesoblastic nephroma (8), and papillary thyroid cancer (9).

Paediatric NTRK rearrangement-related tumours differ from adult counterparts in The epidemiology, histology, molecular, and biological characteristics of pediatric NTRK rearrangement-related tumors differ from those of their adult counterparts. The estimated incidence of NTRK fusion in pediatric tumors is approximately 2%. In general, these tumors have favorable outcomes, with 3-year overall survival (OS) rates > 90%. However, some tumors may recur or metastasize, especially those with high-grade histology or unfavorable sites (10).

Larotrectinib is a potent and orally available, highly selective small-molecule inhibitor of TRK (11). Its oral administration is more convenient compared to other similar therapies which require intravenous infusions at the hospital, thus contributing to a higher compliance, which is a better option for children. Recent phase 1/2 trials have demonstrated high overall response rate (ORR), sustained durable responses and favorable tolerability in different TRK fusion-positive solid tumors (12–14). The objective response rate (ORR) of these agents in pediatric patients ranges from 57.7% to 100% (12). However, data on larotrectinib in children with *NTRK*-rearranged spindle cell tumor are still scarce in China.

Herein, we report four cases of *NTRK*-rearranged spindle cell tumors in China, each harboring distinct TRK fusions achieved both durable response and good tolerability to larotrectinib.

## 2 Case description

**Figure 1:**
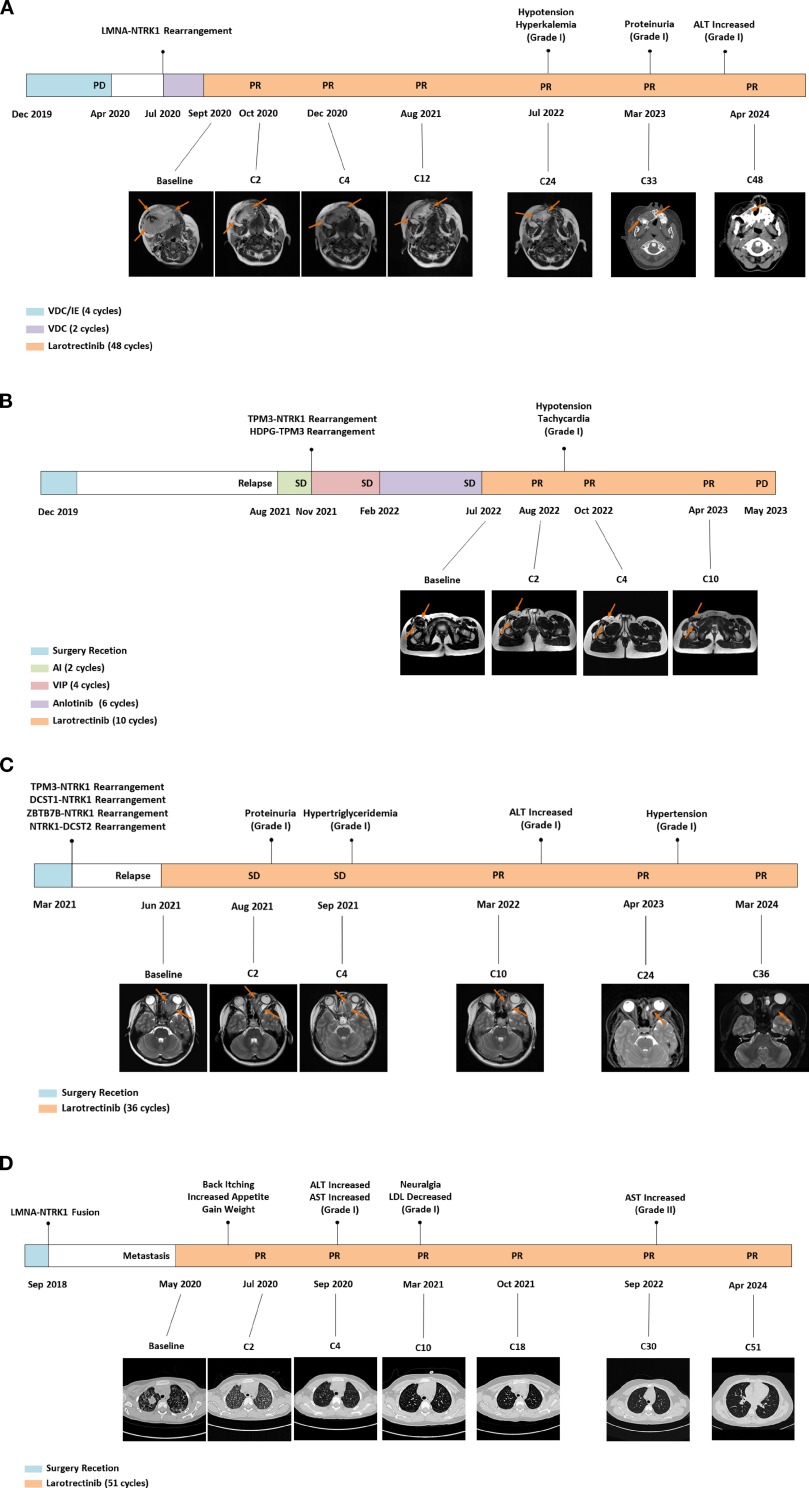
Diagnosis and therapeutic journey, imaging evaluations, genetic testing, and TRAEs of the four patients diagnosed with *NTRK*-rearranged spindle cell tumors. TRAEs, treatment-related adverse events; PD, progressive disease; PR, partial response; MR, magnetic resonance; VDC, vincristine-doxorubicin-cyclophosphamide; IE, isocyclophosphamide-etoposide; ALT, alanine aminotransferase; SD, stable disease; AI, pirarubicin-ifosfamide; VIP, etoposide-ifosfamide-cisplatin; CT, computed tomography; AST, aspartate aminotransferase; LDL, low-density lipoprotein.

Four cases of pediatric patients with TRK fusion-positive solid tumors treated with larotrectinib are presented (**Figure 1**, **Table 1**). *NTRK* fusions were identified via histopathology, immunohistochemistry, and whole-exome sequencing. Larotrectinib was administered as a second- or later-line therapy at 100 mg/m^2^ twice daily. The last follow-up was in April 2024. Patient #1 (facial mesenchymal tumor, *TP53*-*NTRK1*) received four cycles of alternating chemotherapy with vincristine-doxorubicin-cyclophosphamide plus ifosfamide-etoposide, but the disease progressed. Second-line larotrectinib induced partial response (PR) for 48 months, with a tumor regression rate of 44%. Patient #2 (spindle cell tumor in the thigh, *TPM3*-*NTRK1*), who relapsed after tumor resection, achieved PR with larotrectinib for 10 months with a tumor regression rate of 66%. Resistance developed due to a novel *HDGF-TPM3* fusion gene, and subsequent treatment with ICP-723 (a second-generation TRK inhibitor) revealed tumor shrinkage. Patient #3 (retro-orbital spindle cell tumor, *TPM3-NTRK1*, *DCST1-NTRK1*, *ZBTB7B-NTRK1*, and *NTRK1-DCST2*) had local recurrence after surgery and achieved PR with larotrectinib for 36 months, with a tumor regression rate of 43%. Patient #4 (spindle cell tumor in the knee, *LMNA-NTRK1*) had multiple metastases 2 years after surgery and achieved PR with larotrectinib for 51 months. Treatment-related adverse events (TRAEs) in all four cases were grade 1, except one grade 2 aspartate aminotransferase elevation (**Table 1**). No serious TRAEs occurred. There was no treatment interruption or discontinuation due to TRAEs.

**Table 1:** Literature review of children treated with larotrectinib for solid tumors.

Reference	Age/sex	Diagnosis	Fusion gene	Duration of treatment	Dosage	Response	AE	Follow-up
Baranov 2022 (14)	12 years, male	Mesenchymal tumor	TPM3-*NTRK1*	8.8 months	NR	SD	NR	61.3 months, alive
Baranov 2022 (14)	0 month, female	Mesenchymal tumor	ETV6-*NTRK3*	23.6 months	NR	PR	NR	29.3 months, alive
Baranov 2022 (14)	9 years, female	Mesenchymal tumor	ETV6-*NTRK3*	7.2 months	NR	PR	NR	13.5 months, alive
Case #1	4 years, male	Mesenchymal tumor	TP53-*NTRK1*	48 months	100 mg/m^2^ bid	PR	All grade 1: hypotension, hyperkalemia, proteinuria, and elevated ALT.	53 months; alive.
Case #2	11 years, male	Spindle cell tumor	TPM3-*NTRK1*	10 months	100 mg/m^2^ bid	PD	All grade 1: Hypotension and tachycardia.	53 months; alive
Case #3	14 years, male	Spindle cell tumor	TPM3-*NTRK1*, DCST1-*NTRK1*, ZBTB7B-*NTRK1*, and *NTRK1*-DCST2	36 months	100 mg/m^2^ bid	PR	All grade 1: proteinuria, hypertriglyceridemia, increased ALT, and hypertension.	39 months; alive.
Case #4	5 years, male	Spindle cell tumor	LMNA-*NTRK1*	51 months	100 mg/m^2^ bid	PR	Grade 1: neuralgia and decreased LDL. Grade 2: elevated AST.	70 months; alive.

PR, partial response; CR, complete response; NR, not reported; SD, stable disease; PD, progressive disease.

## 3 Discussion

Larotrectinib is a first-generation NTRK inhibitor indicated for patients with TRK fusion-positive solid tumors, regardless of age, tumor type, or fusion partner (12–14). The data on larotrectinib use in pediatric patients with *NTRK*-rearranged spindle cell tumors are still scarce in China. Here, we reported four cases under diverse clinical scenarios: second-line treatment after progressive disease (PD) (case #1), relapse after resection (cases #2 and #3), and metastatic disease (case #4). Larotrectinib treatment led to rapid and durable clinical responses. The longest treatment duration reached 51 cycles, and three patients maintained tumor control at the last follow-up. Notably, Case #2 developed disease progression after 10 cycles of larotrectinib, accompanied by the newly emergence of a novel HDGF-TPM3 fusion gene. Additionally, our cases exhibited distinct NTRK fusion profiles: *DCST1-NTRK1*, *ZBTB7B-NTRK1*, and *NTRK1-DCST2* (Case #3)—all of which have not been previously reported in the literature. These findings expand the evidence base for larotrectinib’s clinical application in this patient population.

Larotrectinib has demonstrated robust efficacy in adult patients with TRK fusion-positive sarcomas, with a reported ORR of 58% and a median progression-free survival (PFS) of 28.3 months, the most common fusion partners named LMNA::NTRK1 and ETV6::NTRK3 (15). Recine et al. described a young adult with a rare *TPM4-NTRK1* fusion-positive spindle cell neoplasm, who experienced rapid and durable regression of both visceral and bone metastases just 7 days after initiating larotrectinib, with no drug-related toxicities observed (16). Then, larotrectinib continues to durable responses with favorable safety in pediatric TRK fusion solid tumors (12, 17, 18). A global multicenter clinical trial further evaluated the safety and efficacy of larotrectinib in 91 pediatric sarcoma patients (including 21 with spindle cell tumors) with an ORR of 87%. Fifty percent of patients experienced treatment-related adverse events (TRAEs) of maximum grade 1 or 2, with no serious adverse reactions observed (17). Most TRAEs of larotrectinib are mild and tolerable, and common TRAEs include electrolyte disturbances, elevated liver enzymes, and blood pressure fluctuations. Among our four cases, all observed TRAEs were grade 1, except for one patient who developed grade 2 aspartate aminotransferase elevation. Notably, Case #4 experienced multiple neurological adverse eventsduring treatment, including back pruritus, increased appetite, weight gain, and persistent yet tolerable neuralgia. These neurological changes are likely attributable to disruption of TRK pathway signaling. When combined adults with the pediatric cases, these reports highlight the tumor-agnostic activity of TRK inhibition in diverse age groups and clinical disease settings.

Management of *NTRK*-rearranged spindle cell tumors requires a multidisciplinary approach, combining surgery with other modalities (e.g., radiotherapy, targeted therapy). However, the optimal timing and duration of TRK inhibitor therapy remain unclear. The risk of disease recurrence following TRK inhibitor discontinuation may depend on multiple factors, including NTRK fusion type, tumor histological grade, extent of surgical resection, and presence of residual disease. A Children’s Oncology Group (COG) study suggested that children with infantile fibrosarcoma (IFS) who achieved complete response (CR) with larotrectinib could discontinue treatment after 6 months without compromising prognosis (19). However, this recommendation may not extend to *NTRK*-rearranged spindle cell tumors. Notably, approximately one-third of patients who electively discontinued larotrectinib experienced disease relapse or progression; yet, 94% of these patients regained disease control upon reinitiating larotrectinib, with 69% achieving an objective response (17). An ongoing prospective COG trial (ADVL1823; NCT03834961) is evaluating response durability following larotrectinib discontinuation in pediatric solid tumors. These studies illustrated the complexity and diversity of clinical scenarios. Longer follow-up and individualized, closely monitored treatment decisions are therefore needed to determine the optimal larotrectinib treatment duration.

Mechanisms of acquired resistance to TRK inhibition are not completely understood. Point mutations in the kinase domain of TRK was the main resistance mechanisms to first-generation TRK inhibitors (6). Few literature reported details of the occurrence of NTRK fusion gene types in pediatric tumors. In our cohort, Case #2 developed larotrectinib resistance after 10 months of disease control, which was associated with the *de novo* emergence of the *HDGF-TPM3* fusion gene. An adult patient with *TPM4-NTRK1* fusion exhibited a durable response to larotrectinib (16). This finding suggests that different *NTRK* fusion partners may affect the efficacy of larotrectinib and the risk of developing resistance to them. Thus, it is essential to identify variations in fusion partners through comprehensive molecular testing and formulate individualized targeted therapy strategies to maximize the therapeutic benefits of TRK inhibitors. *HDGF* is a heparin-binding growth factor overexpressed in various cancers, where it promotes tumor growth, invasion, angiogenesis, and metastasis. *TPM3*—a tropomyosin family gene—encodes a cytoskeletal protein involved in muscle contraction and cell motility. Notably, *TPM3* is a common fusion partner of *NTRK1* in spindle cell tumors; fusion with *HDGF* may activate alternative signaling pathways or augment tumor aggressiveness. Further studies are necessary to delineate the potential mechanisms of *HDGF-TPM3*-mediated resistance.

The small sample size of this study limits the reliability of our findings. Additionally, our exploration of the underlying resistance mechanisms is insufficient, and future studies including larger cohorts and prospective designs are needed to validate these observations.

In summary, we reported the efficacy and safety of larotrectinib in four pediatric cases of *NTRK*-rearranged spindle cell tumors, and discussed the challenges in determining the optimal treatment duration. Notably, *HDGF-TPM3* fusion gene may contribute to acquired resistance to larotrectinib. Further research is needed to explore the potential of second-generation TRK inhibitors for overcoming resistance.

## Data Availability

The original contributions presented in the study are included in the article/supplementary material. Further inquiries can be directed to the corresponding author.
